# Development of a chronic kidney disease patient navigator program

**DOI:** 10.1186/s12882-015-0060-2

**Published:** 2015-05-03

**Authors:** Stacey E Jolly, Sankar D Navaneethan, Jesse D Schold, Susana Arrigain, Victoria Konig, Yvette K Burrucker, Jennifer Hyland, Priscilla Dann, Barbara H Tucky, John W Sharp, Joseph V Nally

**Affiliations:** Department of General Internal Medicine, Medicine Institute, Cleveland Clinic, 9500 Euclid Avenue- G10, Cleveland, OH USA; Department of Nephrology and Hypertension, Glickman Urological and Kidney Institute, Cleveland Clinic, Cleveland, OH USA; Department of Quantitative Health Sciences, Cleveland Clinic, Cleveland, OH USA; Health Informatics Program, Kent State University, Kent, OH USA

**Keywords:** Chronic kidney disease, Patient navigator, Patient education

## Abstract

**Background:**

Chronic Kidney Disease (CKD) is a public health problem and there is a scarcity of type 2 CKD translational research that incorporates educational tools. Patient navigators have been shown to be effective at reducing disparities and improving outcomes in the oncology field. We describe the creation of a CKD Patient Navigator program designed to help coordinate care, address system-barriers, and educate/motivate patients.

**Methods:**

The conceptual framework for the CKD Patient Navigator Program is rooted in the Chronic Care Model that has a main goal of high-quality chronic disease management. Our established multidisciplinary CKD research team enlisted new members from information technology and data management to help create the program. It encompassed three phases: hiring, training, and implementation. For hiring, we wanted a non-medical or lay person with a college degree that possessed strong interpersonal skills and experience in a service-orientated field. For training, there were three key areas: general patient navigator training, CKD education, and electronic health record (EHR) training. For implementation, we defined barriers of care and created EHR templates for which pertinent study data could be extracted.

**Results:**

We have hired two CKD patient navigators who will be responsible for navigating CKD patients enrolled in a clinical trial. They have undergone training in general patient navigation, specific CKD education through directed readings and clinical shadowing, as well as EHR and other patient related privacy and research training.

**Conclusions:**

The need for novel approaches like our CKD patient navigator program designed to impact CKD care is vital and should utilize team-based care and health information technology given the changing landscape of our health systems.

**Electronic supplementary material:**

The online version of this article (doi:10.1186/s12882-015-0060-2) contains supplementary material, which is available to authorized users.

## Background

The recognition, care, and education of patients with chronic kidney disease (CKD) are an emerging public health problem. Current estimates are that more than 26 million adults in the United States have CKD [1]. Patient awareness of CKD is distressingly low [2-4]. Health disparities are well documented in the general population and among the CKD population [5-7]. A more team based approach similar to what is outlined in the chronic care model and patient centered medical home has been hailed as an option to improve patient outcomes and population health [8-11]. Improved primary care provider awareness of CKD and more co-management by primary care and nephrology is also needed [12,13], and an approach that empowers patients and those that support them is desirable. Patient navigators have been incorporated as part of the team and shown to be effective at reducing disparities and improving outcomes in the oncology field [14,15].

A patient navigator is an individual whose primary responsibility is to provide personalized guidance to patients as they interact with and move through health care systems. Dr. Harold P. Freeman, a surgeon in Harlem created the first patient navigation program in the 1980s focused on improving outcomes in breast cancer and reducing disparities through increased screening and complete follow up of abnormal mammograms [14]. Since then there has been over a decade of use in oncology, including multi-year large National Cancer Institute funded clinical trials which found a positive impact of patient navigation on cancer outcomes related to screening, follow-up, and treatment of various common cancers like breast, colorectal, and cervical [15-17]. Consequently, under a new requirement for accreditation by the American College of Surgeons Commission on Cancer, cancer centers must provide patient-navigation services by 2015 [18].

We knew that patient navigation had been quite successful among patients at risk for or with cancer and early evidence suggested it could be translated into similar success for chronic diseases such as cardiovascular disease risk factor reduction or post-stroke care [19,20]. For kidney disease, the use of kidney transplant recipients trained to be patient navigators improved the number of steps patients achieved towards getting on a kidney transplant list [21]. Additionally, a recent study looked at self-identified patient navigator programs spread throughout the United States with use of lay navigator programs more likely in underserved communities representing a potentially important resource in these communities to address disparities and improve quality of care [22].

**Figure 1:**
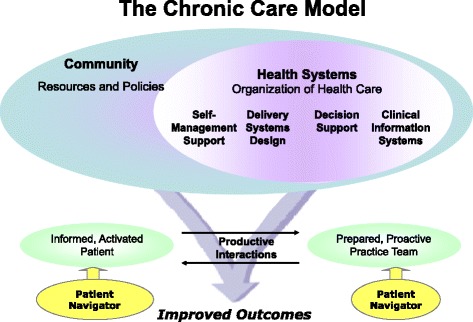
Chronic Care Model [25].

We were one of 5 institutions awarded a grant to pilot translational CKD interventions [23]. We herein describe the design and creation of our CKD Patient Navigator program with a goal to improve outcomes for CKD Stage 3b/4 patients to be recruited from our electronic health record (EHR) CKD registry [24] for a pilot randomized controlled trial (RCT). The conceptual framework for the CKD Patient Navigator Program was to have a more proactive prepared patient and care team in alignment with the principles of the chronic care model [25] [Figure 1].

## Methods

### CKD Patient navigator program

**Figure 2:**
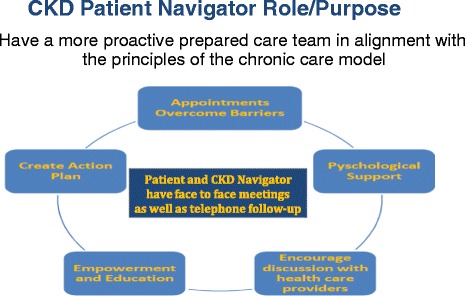
CKD Patient Navigator Role.

Patient navigators do not need to have a medical background; they can be trained to work with patients at every step of the healthcare journey. They can identify and help overcome health system and individual patient level barriers to care, provide health education, work to increase self-management skills, improve coordination of care, facilitate communication among members of the health care team, and provide psychosocial support. [Figure 2].

We believed the key to a successful program would be finding and hiring the *right* person in order to supplement the healthcare team. Based on review of the literature and discussion with personnel who had run other patient navigators programs in our community, we thought the ideal candidate would be a non-medical or lay person with a college degree that possessed strong interpersonal skills and experience in a service-orientated field. Additionally they would need to be a good listener, engage patients to be their own healthcare advocate, have empathy and compassion, a problem-solver, and be dedicated. Finally they would need to be proficient with computers given our health system uses an integrated EHR. The study coordinator and three of the principal investigators were involved with the creation of the job description, reviewing of applications, and interviewing all candidates.

**Figure 3:**
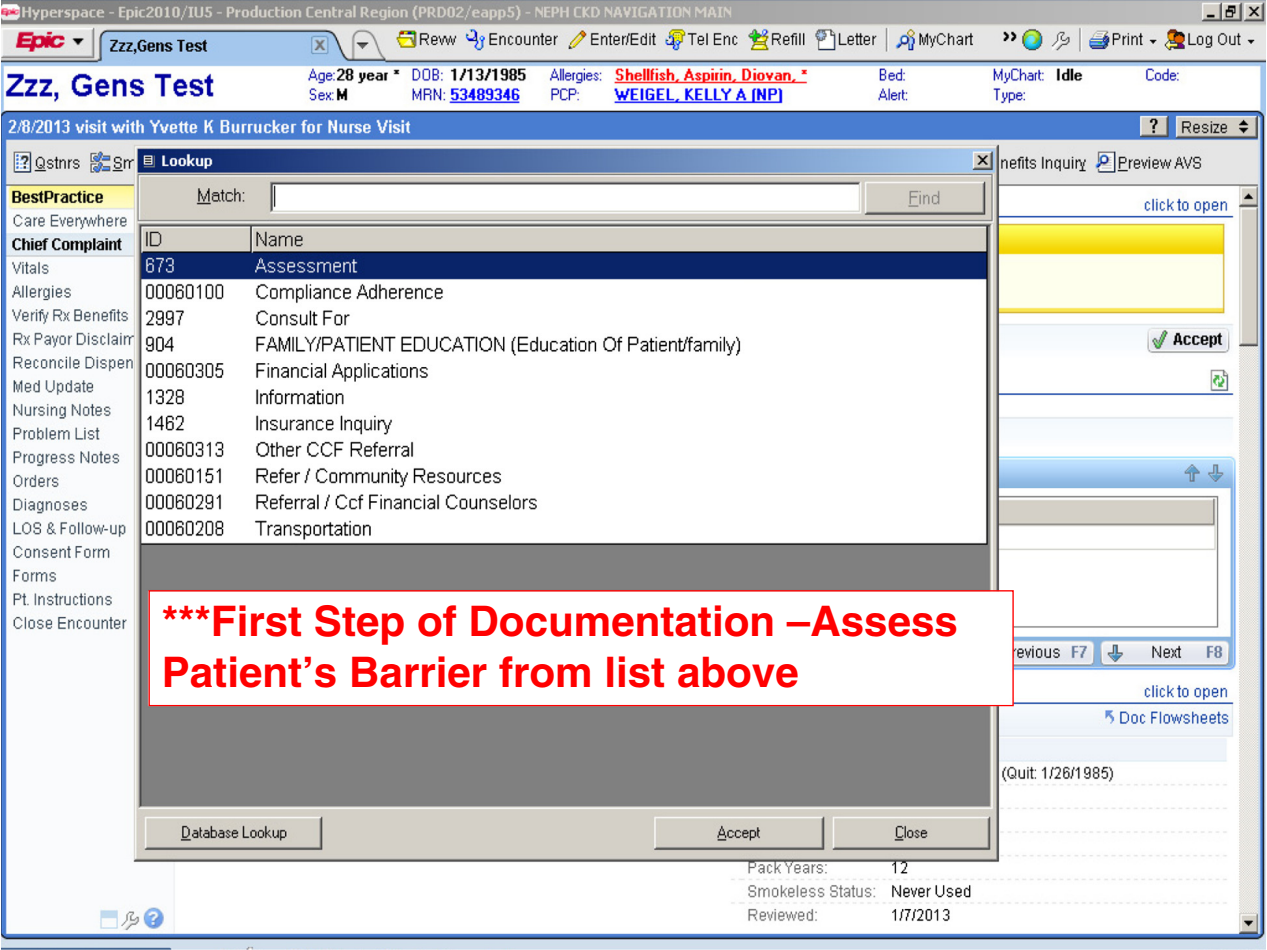
CKD Patient Navigator EHR Template.

Furthermore, as a team, we defined *a priori* barriers of care based on what had been published in the oncology literature and what we thought would be important to track for patients in our health system [16,26-28]. We created EHR templates for the CKD patient navigators to use for which pertinent study data on barriers could be extracted but more importantly would also serve as communication to the health care team members. We agreed that patient navigators would document in the EHR their face to face visits as well as telephone encounters with patients. The EHR templates were created by our health information technology personnel and they were tested using computer test patients by the study team. Changes to the template were made through an iterative process before implementation. The final EHR templates allowed for some patient and clinical data to automatically be included into the note and key study metrics to be documented but recognized the need for freedom of patient navigators to be able to document the summary of their visit [Figure 3].

We designed the training to be similar to what prior patient navigators have completed but also included specifics related to CKD that would be important when implementing a CKD patient navigator program. The training we developed consisted of three key areas: general patient navigator training, CKD education, and EHR training.

#### General patient navigator training

We wanted to make use of a structured training program and found that with The Harold P. Freeman Patient Navigation Institute [29]. Established in 2007, The Freeman Patient Navigation Institute offers a paid intensive online patient navigator certification program; upon completion of the online program within 30 days the navigator receives a certificate. The curriculum is comprehensive and interactive, includes practical experience along with best practice research. Though the Freeman Patient Navigation Institute curriculum focuses on oncology, they state it can be adapted to other fields. We saw the patient navigator approach as adaptable for CKD in that it is important to define your population, follow the population through time, and have a plan to address barriers of care and create action plans to address needed disease management.

#### CKD education

Given that the patient navigators would have a non-medical background but would be working with patients with CKD, we incorporated general CKD education into the training. CKD education was provided by directed readings, online via the National Kidney Disease Education Program (NKDEP) and The National Kidney Foundation (NKF) websites educational materials. We selected these sites as they are publically available and have a breadth of educational materials that are easy to read and/or had good visual images. We chose specific educational materials covering general knowledge of what is CKD to more detailed educational materials regarding choosing dialysis and information on fistula placements [See Additional file 1].

To supplement the readings with real world experience we asked that patient navigators shadow Certified Nurse Practitioners (CNP) in the CKD clinic as part of their training for a total of four sessions. The CKD clinic shadowing sessions were anticipated to last approximately 4 hours each allowing adequate time for observation of interactions between the CNP and patient that were focused on CKD specific topics such as important CKD symptoms, nutrition, preparing for dialysis or transplant referral, communication with other healthcare team members, and local resources.

#### EHR training

Our health system uses the integrated EHR software system, EPIC© (Madison, WI). Since CKD patient navigators would be interacting directly with patients, accessing and documenting in the patients’ EHR, EPIC© training was essential. The interactions between patient navigators and patients we planned to be documented not only for the purpose of the study but for the entire healthcare team involved in the care of the patient as well. Cleveland Clinic has ongoing EHR training via online modules available to all new hires. Protection of Human Subjects through Collaborative Institutional Training Initiative (CITI) and Health Insurance Portability and Accountability Act (HIPPA) training were required as well. EHR, CITI, and HIPPA training were anticipated to take about one week to complete either online or via in-person classes.

This study was approved by Cleveland Clinic Institutional Review Board (CCF IRB# 12-072).

## Results/Lessons learned

### CKD patient navigator program

#### CKD navigator hiring

The job description for the CKD patient navigator position was advertised through the Cleveland Clinic jobs website. We hired our first full-time patient navigator March 2012 and our second part-time patient navigator April 2013. Key lesson learned was selecting among the applications a person with initiative to be a part of the process developing a new program, be flexible with ambiguity, and yet be resourceful and collegial. The job requires a person be capable of working closely with administration, physicians, other caregivers, and patients and their families. One of the patient navigators has a human resources background and the other has a library sciences and health advocacy background. Each had the appropriate personal and computer skills outlined to meet the needs of the program. Salary was commensurate with experience but considerably less than a registered nurse or CNP; in alignment with a goal of team-based care which is to have people practicing at the top of their license. One of our CKD patient navigators was hired to work full time and follow 70 patients and the other was hired to work part-time and follow 32 patients.

#### CKD patient navigator training

We wanted our patient navigators to have their certificates through Freeman Patient Navigation Institute [29]. Our first CKD patient navigator completed this online course after being hired. It was completed in approximately 36 hours over a 30-day period. The certification program consisted of five modules. At the end of each module was an assessment test for which you needed a 70% or higher pass rate to move onto the next module. The second patient navigator hired was already certified through the Freeman Patient Navigation Institute.

Their EPIC©, HIPPA, and CITI training took place through already established mechanisms for new hires at Cleveland Clinic and was completed in two days. However additional specific training and troubleshooting particular for the electronic health record was identified given this was a new program being implemented. CKD patient navigator templates had to be created and tested to make sure would suit not only the research needs for data extraction but also be useable by the patient navigators and other team members for patient care. This required interdisciplinary meetings with an EPIC© clinical analyst, study coordinator, patient navigator, research team members representing nephrology and primary care, and statisticians. It took approximately 6 meetings over a one month process to get a working CKD patient navigator EPIC© template.

They each spent four days in CKD clinic with our CNPs observing provider-patient interactions as well receiving CKD-specific education. Through this clinical observational experience, patient navigators were able to witness the ways in which the CNP educated the patient on the importance of recognizing their own medications, managing their own illness proactively and the need for patient participation in their healthcare. The CNP educated the patient navigator regarding local resources available for patients for example to assist with medication costs, transport costs or assistance with utilities. As well, CKD patients may need short or long term disability during the course of their illness so this was discussed. The patient navigator observed and took note of the key concerns that are focused on at every visit such as edema, chest pain, increasing shortness of breath or changes in urination or appetite. The patient navigator was made aware when to ask the patient to contact the provider if there has been a change to the patient’s conditions or complaints. The goals of the shadowing sessions were to make the patient navigator aware of the complexity of CKD, chronic disease management, and patients themselves so they may provide guidance and work with the patient and team to achieve the best possible outcome for the patient.

#### CKD patient navigator barriers

A set of ten barriers or categories were defined *a priori* by the team to allow for consistency and future tracking of the impact of patient navigators. Barriers were determined by review of the oncology literature and then discussion by the team on what health system specific barriers would be important to assess. As well the team identified the need to allow for flexibility in the form allowing for a free text or other category for which additional undefined barriers might be discovered. The *a priori* barriers or categories were: assessment, compliance/adherence, consultation for, education of patient or family, financial applications, information, insurance inquiry, refer to Cleveland Clinic financial counselors, other Cleveland Clinic referral, community resources, and transportation. In addition to selecting at least one of these barriers, patient navigators can free text documentation of other barriers identified and addressed at that visit. The list of barriers and how they might be addressed by the CKD patient navigators is listed in Table 1.

**Table 1:** **List of barriers and example of how they might be addressed by a CKD patient navigator**

**Barrier**	**Example of how it might be addressed**
Assessment	First meeting with the patient; assess areas the patient is interested in working on, determine best mode of future communication, and set up the next meeting
Compliance/Adherence	Patient reports problems obtaining medications due to costs; discuss with patient to find out if there are lower cost medications through their formulary or determine if there are pharmaceutical patient assistance programs the patient might qualify for and assists with obtaining any necessary applications.
Consultation for	Patient recommended to see a specialist but hasn’t made the appointment; patient navigator helps to facilitate appointment by contacting speciality department and coordinating appointment that is right for them based on their location needs or other factor.
Education of patient/family	Edudate patients and/or their family members who do not have knowledge about CKD with resources available to them from NKF, NKDEP, National Diabetes Foundation, and Cleveland Clinic’s patient and family health education center.
Financial applications	Patient reports difficulty with utility bills; patient navigator helps contact appropriate agencies for assistance.
Information	Help them get the basic information needed to navigate the Cleveland Clinic’s large health system. This could include directions, parking information, making sure they have contact numbers for their physicians.
Insurance inquiry	Patient reports billing issues; patient navigator helps coordinate communicates with billing department about available options the patient qualifies for to obtain assistance with bills.
Refer to CCF financial counselors	Patient reports having no health insurance; referral to our financial counselors who helps determine what available health programs the patient qualifies for to obtain health insurance and or assistance.
Other CCF referral	Assists patients with how to acquire medical equipment (e.g.. continuous positive airway pressure machine and mask) that was prescibed to them for sleep apnea.
Community resources	Patient is elderly, lives alone, and would like to remain independent; identify community resources for that senior in his/her city, for example, housing that has both independent and assistance living options if patient needs it at a later date and community exercise programs that offer free to low-cost exercise classes and programs.
Transportation	Patient reports he or she is having transportation issues getting to appointments; patient navigator assists with completion of local public transportation application and coordinates with medical provider for signature approval.

CKD = Chronic Kidney Disease.

NKDEP = National Kidney Disease and Education Program.

NKF = National Kidney Foundation.

#### CKD patient navigator-patient meetings

We predetermined there would be an initial face-to-face meeting between the CKD patient navigator and the CKD patient that had been enrolled and randomly assignment to the navigator arm of the clinical trial. At that meeting, identification of barriers, goals of care, and an action plan might be made with the CKD patient navigator. Communication preference could be established (e.g. telephone, e-mail); *CKD patient navigator-patient* visits were planned at a minimum to occur on a monthly to quarterly interval based on CKD stage; in addition the patient navigators would be available as needed. We wanted the meetings to take place at a Cleveland Clinic building, such as a clinic or family health center and ideally capitalized on a date and time for which the patient is already coming for a regularly scheduled appointment. To allow for flexibility, we predetermined those subsequent planned meetings could also take place via the phone or in person depending on the schedule of the patient and/or patient navigator.

Some lessons learned identified by the patient navigators were logistics, importance of building trust, and maintaining effective communication/engagement. For logistics, meeting locations between patients and patient navigators had to be arranged in advance and varied depending on the location within our health system. This required working with local administration of where the meeting was to take place to identify an available location such as a clinic room, private space in a lobby, or in an education conference room. For building trust, a telephone script was created by the patient navigators for introduction and setting up of the first face to face meeting. Then at that meeting establishing initial rapport and expectations was crucial as this was a new relationship within the context of health for not only the patient but the patient navigator as well. Lastly, effective communication and maintaining of patient engagement in their health was ascertained as essential because of the need to illicit barriers, work together as a patient/patient navigator team but also with other healthcare team members to help address them, and just the nature of managing chronic disease over time.

## Discussion

Healthcare is undergoing tremendous transformation and there is a need for us to address CKD, its risk factors, and its complications, in unique innovative ways. There is optimism among the kidney community of researchers, clinicians, and patients that translational research will lead to improved outcomes [30]. Strategic use of EHRs to help tackle the challenges associated with CKD will be needed [31]. Additionally, a recent review of health information technology and CKD publicized anticipation that it will lead to improved CKD care too [32]. We described the creation of one novel educational intervention with a goal of improving the care of CKD patients and addressing a paucity of type 2 translational research [23]. The CKD patient navigator program was developed to empower/educate patients and their care team through the acquisition of skills based on patient navigator experience in the oncology field but with important adaptation for CKD and the chronic nature of the condition.

Even with the widespread implementation of several education programs, lack of patient awareness of CKD persists [33,34]. While routine reporting of the estimated glomerular filtration rate (eGFR) has been advocated as a means of helping primary care providers identify CKD earlier, routine reporting alone has not been sufficient to improve CKD outcomes [35]. Evidence exists for improvement of outcomes and decreased health care utilization among patients with chronic disease who participate in disease self-management programs [36-38]. Action planning seems to be a key factor of self-management interventions [39]. Whether such chronic disease self-management programs will lead to success among patients with CKD was inconclusive based on a recent systematic review [40].

One of the challenges in the development of the CKD Patient Navigator Program was the very limited experience of using patient navigation for conditions other than cancer. There was evidence that lay patient navigators at an urban safety-net health care system who were trained in community health and peer counseling with a goal of reducing cardiovascular risk did find improvement in some health behaviors at one year [20]. Specifically for kidney disease, a recent randomized clinical trial hired and trained study coordinators who were kidney transplant recipients themselves to act as navigators and were able to help patients eligible for kidney transplant achieve more of the steps towards being listed on a transplant waiting list [21]. However, there is no set standard for what patient navigation is in chronic disease. We sought to learn from our cancer colleagues their experience with regards to metrics and measurement for evaluation of patient navigators [28,41]. We plan to share our experience with the predefined metrics as well those that maybe discovered through the process at the completion of the planned RCT [23].

## Conclusions

In summary, the need for new educational approaches to positively impact CKD care is imperative and will require team-based care and health information technology given the changing landscape of our health systems. In oncology, patient navigation has developed from its roots as a way to address the disparities of identification and treatment to multi-center, multi-year NIH-funded clinical trials to scientifically test the efficacy of patient navigation to it now being the standard of care for most cancer programs. Our intent was to learn from this experience and aspire to translate analogous success into the CKD world with the creation of a CKD patient navigator program.

## Additional file

Additional file 1:
**CKD Educational Resources.**

## Abbreviations

CKD: Chronic kidney disease
EHR: Electronic health record
CNP: Certified nurse practitioner
CITI: Collaborative Institutional Training Initiative
HIPPA: Health Insurance Portability and Accountability Act
RCT: Randomized control trial
