# Supplementary material for: Development of a chronic kidney disease patient navigator program
Source: BMC Nephrol. 2015 May 3;16:69. doi: 10.1186/s12882-015-0060-2 (PMC4459709; doi:10.1186/s12882-015-0060-2)
Supplement: Additional file 1: — CKD Educational Resources. [file 12882_2015_60_MOESM1_ESM.doc]

CKD Resources

General Information

What is Chronic Kidney Disease?

<http://www.kidney.org/kidneydisease/>

What does Chronic Kidney Disease mean for me?

<http://nkdep.nih.gov/resources/CKD_Basics_brochure.htm>

Tests for Chronic Kidney Disease

Testing for kidney disease

<http://nkdep.nih.gov/learn/testing.shtml>

What is GFR (glomerular filtration rate)?

<http://nkdep.nih.gov/learn/testing/understand-gfr.shtml>

What is albuminuria (protein in the urine)?

<http://www.kidney.org/atoz/content/albuminuria.cfm>

Managing your Chronic Kidney Disease

Talking with your doctor about your kidney numbers

<http://nkdep.nih.gov/living/working-with-providers.shtml>

Track your kidney test numbers

<http://www.nkdep.nih.gov/resources/nkdep-kidney-test-results-508.pdf>

Medications what to avoid when you have Chronic Kidney Disease

<http://nkdep.nih.gov/resources/CKD_Medicines.htm>

Nutrition—How to read a food label

<http://nkdep.nih.gov/resources/NKDEP_NutritionFactsheets_FoodLabel_508.pdf> Nutrition—Eating Right

<http://nkdep.nih.gov/resources/eating-right.shtml>

Staying Fit With Kidney Disease

<http://www.kidney.org/atoz/content/stayfit.cfm>

Common conditions with Chronic Kidney Disease

High Blood Pressure and Chronic Kidney Disease

<http://www.kidney.org/sites/default/files/docs/hbpandckd.pdf>

Diabetes and Chronic Kidney Disease

<http://www.kidney.org/atoz/content/diabetes.cfm>

Cholesterol and Chronic Kidney Disease

<http://www.kidney.org/atoz/content/bloodlipids.cfm>

Obesity (Body Mass Index ≥ 30)

<https://www.kidney.org/atoz/content/obesewyska>

National Resources

National Kidney Foundation (NKF)

<http://www.kidney.org/>

National Kidney Disease Education Program (NKDEP)

<http://nkdep.nih.gov/>

PubMed Health

<http://www.ncbi.nlm.nih.gov/pubmedhealth/PMH0001503/>

Local Resources

Cleveland Clinic Chronic Kidney Disease Guide

<http://www.clevelandclinic.org//lp/chronic-kidney-disease/index.html?utm_campaign=CS+-+Urology+-+DR+-+CKD&utm_medium=cpc&utm_source=googleppc&utm_term=chronic+kidney+disease&002=2107636&004=2214769962&005=264883523&006=9059511762&007=Search&008=&gclid=CIHChc34yq0CFRFV7AodwCVThg>

Support Groups—Kidney Foundation of Ohio

<http://www.kfohio.org/>

Graduate Level Patient Information

American Association of Kidney Patients

<http://www.aakp.org/>

►Stage 4 (eGFR 15-29)

Complications of Chronic Kidney Disease

Anemia and Chronic Kidney Disease

<https://www.kidney.org/atoz/content/what_anemia_ckd>

Iron and Chronic Kidney Disease

<https://www.kidney.org/atoz/content/iron_ckd>

Nutrition is important in Chronic Kidney Disease

Sodium (salt) and Chronic Kidney Disease

<http://nkdep.nih.gov/resources/nutrition-sodium.shtml>

Potassium (electrolyte) and Chronic Kidney Disease

<http://nkdep.nih.gov/resources/nutrition-potassium.shtml>

Phosphorus (bone health) and Chronic Kidney Disease

<http://nkdep.nih.gov/resources/nutrition-phosphorus.shtml>

Protein (meat, eggs, dairy, fish) and Chronic Kidney Disease

<http://nkdep.nih.gov/resources/nutrition-protein.shtml>

Planning for End Stage Kidney Disease

What is End Stage Kidney Disease?

<http://www.ncbi.nlm.nih.gov/pubmedhealth/PMH0001529/>

Dialysis Overview

<http://www.ncbi.nlm.nih.gov/pubmedhealth/PMH0004676/>

Hemodialysis

<https://www.kidney.org/atoz/content/hemodialysis>

Peritoneal Dialysis

<https://www.kidney.org/atoz/content/peritoneal>

Kidney Transplant

<http://www.ncbi.nlm.nih.gov/pubmedhealth/PMH0003496/>

What if you do not want dialysis?

<https://www.kidney.org/sites/default/files/docs/ifyouchoose.pdf>

What about stopping dialysis if you are already on it?

<https://www.kidney.org/atoz/content/dialysisstop>
